# Enhancing Multichannel Fiber Optic Sensing Systems with IFFT-DNN for Remote Water Level Monitoring

**DOI:** 10.3390/s24154903

**Published:** 2024-07-29

**Authors:** Erfan Dejband, Tan-Hsu Tan, Cheng-Kai Yao, En-Ming Chang, Peng-Chun Peng

**Affiliations:** 1 Department of Electrical Engineering, National Taipei University of Technology, Taipei 10608, Taiwan; t109319413@ntut.edu.tw (E.D.); thtan@ntut.edu.tw (T.-H.T.); 2 Innovation Frontier Institute of Research for Science and Technology, National Taipei University of Technology, Taipei 10608, Taiwan; 3 Department of Electro-Optical Engineering, National Taipei University of Technology, Taipei 10608, Taiwan; t109658093@ntut.org.tw (C.-K.Y.); t110658052@ntut.org.tw (E.-M.C.)

**Keywords:** fiber optic, FSO, IFFT-DNN, water level sensor, remote sensing

## Abstract

This paper proposes a novel approach to enhance the multichannel fiber optic sensing systems by integrating an Inverse Fast Fourier Transform-based Deep Neural Network (IFFT-DNN) to accurately predict sensor responses despite signals overlapping and crosstalk between sensors. The IFFT-DNN leverages both frequency and time domain information, enabling a comprehensive feature extraction which enhances the prediction accuracy and reliability performance. To investigate the IFFT-DNN’s performance, we propose a multichannel water level sensing system based on Free Space Optics (FSO) to measure the water level at multiple points in remote areas. The experimental results demonstrate the system’s high precision, with a Mean Absolute Error (MAE) of 0.07 cm, even in complex conditions. Hence, this system provides a cost-effective and reliable remote water level sensing solution, highlighting its practical applicability in various industrial settings.

## 1. Introduction

Accurate liquid level measurement is essential in numerous industrial applications, such as oil and water reservoir management, fuel storage, and construction monitoring. Over the years, various sensor technologies, such as electrical, mechanical, and electromagnetic-based sensors, have been developed to meet this demand, each presenting its own set of advantages and limitations. However, fiber optic-based sensors show significant advantages compared to conventional ones [1,2,3,4] including electromagnetic anti-interference, low cost, fast response time, high sensitivity, and reduced sensor size. Furthermore, these sensors make remote sensing possible. Since information is conveyed via light signals, the sensing devices can be positioned at remote or difficult-to-reach locations and yet still transmit data to the central monitoring system without experiencing signal deterioration. To overcome this challenge, Free Space Optics (FSO) technology meets the requirements while offering several additional advantages [5,6,7].

FSO is a wireless transmission method that uses laser or infrared beams to transmit data across a free space, similar to fiber optic communication systems but without the need for physical cables. This approach is highly suitable for point-to-point communication, providing a higher bandwidth, faster data rates, low power consumption, and no licensing requirements. Additionally, FSO ensures very high security, immunity to electromagnetic interference, and cost-effectiveness. It can be installed easily and quickly, making it a practical solution. FSO communication enables the systematic transport of large amounts of data in gigabytes, which is not feasible with traditional radio or microwave transmission methods [8,9,10]. Weather conditions such as rain, fog, snow, and heat hazes can significantly impact the transmission quality of FSO channels by causing attenuation and a scattering of the optical signal. Despite these challenges, FSO technology has demonstrated its resilience through the use of advanced error correction techniques and adaptive modulation schemes. For instance, a novel encryption technique has been developed to enhance the performance of FSO communication systems in rainy weather, which not only improves the data security but also mitigates the effects of signal degradation [11]. Also, the presence of birds and other fauna can temporarily obstruct the FSO path, leading to signal interruptions. However, this issue can be effectively mitigated using spatial diversity techniques, where multiple FSO links are established in parallel. This redundancy reduces the likelihood of a complete signal blockage, ensuring more reliable communication. These techniques enhance the robustness of FSO systems against transient obstructions caused by fauna [12]. Furthermore, [13] introduced a bidirectional FSO link with an amplifier to enhance the system’s reliability, improve the signal-to-noise ratio, and reduce the power lost.

Although fiber optic-based sensors offer numerous advantages, interpreting the raw data they produce necessitates distinct techniques and algorithms. Furthermore, the increasing number of sensors brings higher costs and additional equipment, as each sensor requires its own operational bandwidth. Wavelength Division Multiplexing (WDM) and Intensity Wavelength Division Multiplexing (IWDM) are two traditional methods to increase the number of sensors in a sensing system. However, these approaches often result in overlapping and crosstalk between sensors operating within the same bandwidth [14,15,16].

A highly multiplexable method for creating an array of active fiber sensors has been proposed in [17] as well as the process for creating easily multiplexable active fiber sensors. High-temperature stable Type-II fiber sensor arrays are produced in conventional telecom fibers using the femtosecond laser direct writing technique. In the demonstration, six Type-II FBG sensors were used in an active fiber sensor array. These six FBG sensors can be optically heated, enabling both passive and active measurements with a 225 mW in-fiber power. In [18], a dense wavelength division multiplexing (DWDM)-based passive optical fiber sensor network has been demonstrated to remotely monitor the spent fuel pool’s water level in nuclear power facilities. An arrayed waveguide grating (AWG) for DWDM has been employed, and the water level was measured in real time by analyzing the optical signal spectrum relayed from the sensing units. However, this method used several sensors for only measuring a single spot which leads to increased cost when needed to measure multiple spots. A FBG temperature sensor array as a framework for a liquid level measuring and classification system has been explored in [19]. The fluids were divided into two categories for the oil classification: oil and non-oil, or water and emulsion. The random forest (RF) technique was selected for the classification due to the low variability of the classes. In [20], the authors also interrogated several FBG temperature sensors, and several machine learning methods were used to predict the water levels by using nine FBG sensors. However, the prediction error is still more than 7 cm in some cases and more than 3 cm on average. 

Recently, with the aid of machine learning (ML) and deep learning (DL) algorithms, many sensing systems have gained significant advantages in various applications, such as autonomous driving, healthcare monitoring, environmental sensing, and industrial automation [21,22,23]. As an example, in [24] a section of a high-speed train track has been set up with a distributed optical fiber acoustic system (DAS). A novel approach to track detection has been provided by using semi-supervised deep learning based on picture recognition, with a greedy algorithm for the hyperparameter selection. In [25], to improve the accuracy and efficiency of bridge structure damage detection, a deep learning algorithm has been employed while continuously measuring bridge deflection using a fiber optic gyroscope. A deep convolutional neural network-based supervised learning model has been proposed to detect three different damage scenarios. In [26], a deep learning-based method for measuring water levels using YOLOv5s and a convolutional neural network was developed. This method was validated with a video monitoring station and showed a systematic error of 7.7 mm. Despite its advantages, this approach faces challenges when visual scenes are obscured or in environments where the deployment of surveillance systems is prohibited.

In multichannel sensing systems, when sensors multiplex, crosstalk, and experience signal overlapping, it can be challenging to interpret environmental parameters such as temperature, tension, and liquid levels. To address these challenges, several algorithms have been proposed, all of which focus on the frequency domain [21,27]. Recent advances in the graphics processing unit (GPU) and tensile processing unit (TPU) technologies have facilitated the development of more robust neural networks, and among these networks hybrid neural networks have demonstrated several advantages, including enhanced feature learning, flexibility, modularity, and interpretability. In hybrid neural networks, different branches can be specialized to learn distinct types of features or representations. This multi-branch approach allows the model to capture a more diverse set of features, which can be integrated to improve the overall performance. This branching also helps to disentangle the different factors of variation in the data, leading to more interpretable model decisions [28,29]. 

In this paper, we propose a novel dual-domain approach to enhance the sensing system by integrating an inverse fast Fourier transform in a hybrid deep neural network with three branches to accurately predict water levels at multiple points in remote areas. To measure water levels in remote areas we employed the FSO link which offers outstanding advantages due to its high-speed data transmission capabilities without physical cables. The FSO link used in our study covered a distance of 2 m and was tested under controlled laboratory conditions, at room temperature (26 °C), and under artificial and stable light with no wind. In cases where the environment is free from environmental factors or atmospheric turbulence, it is possible to transfer data over a 1 km distance utilizing FSO. Additionally, the FSO link length can be extended to more than 2 km for the commercial industry [30,31,32]. In demanding applications that need sensing across diverse and geographically dispersed locations, the integration of FSO helps to simplify the deployment process and significantly reduces the installation costs [9,33]. Additionally, to measure multiple points cost-effectively we used a multichannel sensing system framework. Furthermore, in this proposed dual-domain approach, we used a hybrid neural network named IFFT-DNN with three branches that use the frequency domain as well as the time domain (real and imaginary part) of the sensors’ signal to extract more features, which leads to more robust and accurate water level predictions. By leveraging the hybrid neural networks and integrating both the frequency and time domain features of the sensor signals, a broader range of patterns in sensor data can be captured, resulting in higher accuracy and precision, even in severe cases of crosstalk. Analyzing both the frequency and time domains ensures a richer representation of the input data, enabling the network to learn more robust and detailed features. The distinct processing of different data components (the frequency domain, real, and imaginary parts of the time domain) in each branch, allows for a better gradient flow during backpropagation which enhances the overall training efficiency. 

## 2. Proposed Structure and Experimental Setup

**Figure 1:**
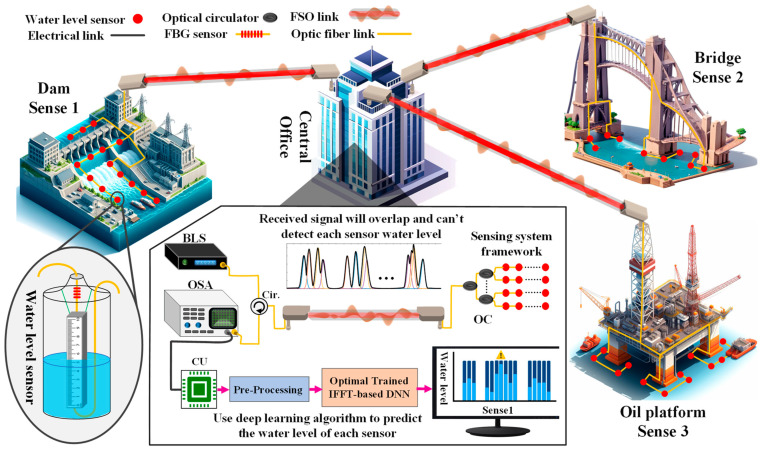
The proposed water level sensing system utilizing FSO links and multichannel fiber sensors enhanced by an IFFT-DNN to predict multi-point water levels at remote areas. The broadband light source (BLS) and optical spectrum analyzer (OSA) are depicted in the box on the left side, and the OC and Cir are the optical coupler and the optical circulator, respectively. Details of the pre-processing part are described further in this paper.

The proposed sensing system is illustrated in Figure 1. To measure the water level of multiple points in remote locations, the broadband pulse from a Broadband Light Source (BLS) is sent through the circulator (Cir.) (in the box under the central office in Figure 1) and FSO link to arrays of Optical Couplers (OC), which direct the light towards the sensors array with different intensities. The reflected spectrum received in the central office is captured by an Optical Spectrum Analyzer (OSA), however, according to the crosstalk between sensors, it is not possible to interpret the water level of each sensor directly. The crosstalk between sensors leads to signals overlapping and creates the multiplet or in severe cases a single peak. Hence, the received signal is fed to the Computational Unit (CU) to apply pre-processing and then to the well-trained IFFT-DNN to predict the water level of each sensor. In the pre-processing part, detailed further in this paper, the received signal was normalized and the IFFT was applied to the signal. Then, the frequency domain along with the real and imaginary parts of the time domain is fed to the well-trained IFFT-DNN and its details are discussed in Figure 1.

The choice of not using an optical switch in the proposed sensing system was deliberate and based on several considerations. The primary goal was to develop a robust and efficient method for remote water level sensing that could handle signal overlapping and crosstalk between multiple Fiber Bragg Grating (FBG) sensors. This method allows the use of numerous sensors on a single optical fiber, significantly increasing the sensing capacity without the additional costs and equipment. While an optical switch could theoretically avoid an overlapping of FBG spectra, it introduces drawbacks, such as delays in data acquisition due to sequential channel selection, which hinders real-time monitoring, especially with many sensors. The proposed method enables simultaneous data acquisition from all sensors, allowing real-time monitoring and faster response times. Additionally, optical switches can be costly, particularly when scaling up systems with many sensors. The proposed method eliminates the need for optical switches, reducing the overall cost and making the system more economically viable for large-scale deployments in remote areas.

As a concrete application example, the proposed method offers significant benefits for bridge monitoring applications. Real-time water level measurements are provided by the sensing system, enhancing the safety and operational efficiency of bridge infrastructure. The accurate monitoring of water levels around bridge piers and abutments is crucial for detecting potential flooding or erosion, which can compromise the structural integrity. Measurement accuracy and reliability are improved by the dual-domain approach of combining the frequency and time domain analyses, ensuring that any changes in water levels are detected promptly. This allows for early warning and preventive maintenance, reducing the risk of bridge failure. Furthermore, its consistent performance makes the system highly suitable for long-term deployment in diverse settings. The ability to monitor multiple sensors simultaneously without significant additional cost to the system also means that large bridge structures can be comprehensively monitored, providing a robust solution for maintaining the safety and longevity of critical infrastructure.

**Figure 2:**
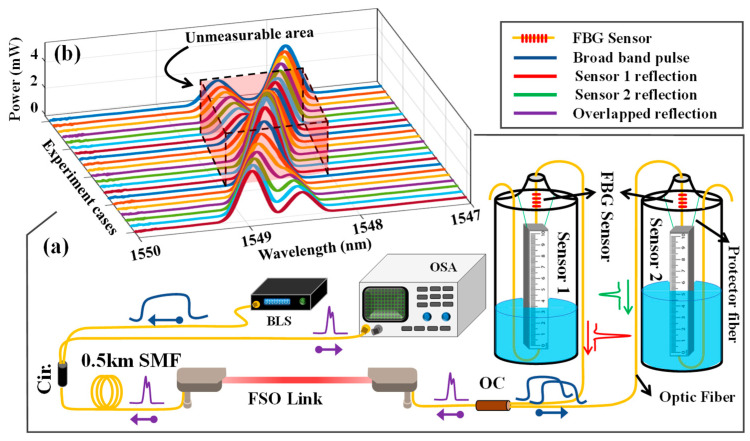
(**a**) The experimental setup. (**b**) OSA singles for different case experiments.

In this paper, we focus on two sensors to investigate the crosstalk and sensor interference in the system. Figure 2a depicts the experimental setup to measure water levels with two CP-900 water level sensors manufactured by Citpo Technologies Inc. (Taipei, Taiwan), in which the two sensors have identical operating wavelengths. The BLS emits a broadband pulse to the optical circulator, preventing any interaction between the emitted and reflected light. The broadband light then transmits through the FSO channel to the 70:30 optical coupler and the sensors. In the experiment, a light source was used to cover the typical range of FBG band operation, which typically is 1528–1568 nm. The extinction ratios for the FBG sensors are 5.25 dB and 3.1 dB, respectively, with a central wavelength of 1548 nm and a standard single-mode fiber utilized. The FSO link utilized in the experimental setup spanned a distance of 2 m in controlled laboratory conditions, maintained at a room temperature of 26 °C, however, it is possible to transfer over a 1 km distance [30].

To measure the water level, each sensor is equipped with a fiber Bragg grating (FBG), which is connected to an indicated float and is well-protected. The float is composed of two securely attached rectangular sections, front and back. The optical fiber is positioned between these sections, with the FBG sensor placed on top of the float in a protected area. This configuration ensures that the FBG sensor is well-shielded from potential damage. Additionally, side protection is incorporated into the float to limit its movement, preventing excessive tension on the FBG and minimizing the risk of damage to the sensor. This design maintains the integrity and functionality of the sensor, allowing for accurate measurements of water level changes. The FBGs are light-reflecting structures that reflect a semi-Gaussian spectrum at specific wavelengths while transmitting the broadband light spectrum. The central wavelength of the semi-Gaussian reflected spectrum, known as the Bragg wavelength (*λ*B), corresponds to the peak wavelength of the reflected spectrum of a single sensor [34].

Changes in the water level cause the float to move, thereby altering the tension along the fiber. This tension induces a shift in the center of the semi-Gaussian reflected spectrum or Bragg wavelength of the FBG sensor. Both the compressive and tensile strain change the fiber length, which in turn modifies the grating period, resulting in a shift in the Bragg wavelength. To investigate the overlapping situation, one water level sensor was filled with ~6 cm of water while the water level in the other sensor changed from 0 cm to 10 cm with steps of 0.5 cm. The reflected spectrum of each case experiment was later captured by OSA, as illustrated in Figure 2b, which depicts all 21 case experiments as well as the unmeasurable area, causing a partial or full overlapping. Additionally, in Figure 2a, the broadband pulse is shown in blue. After being transmitted via the FSO link, it is split by an optical coupler. The reflected spectra of Sensor 1 and Sensor 2, each with different water levels, are shown in red and green, respectively. Also, the total reflection is depicted in purple, which is directed to the OSA by an optical circulator. Based on our system’s design and OSA settings, the span wavelength is 4 nm, the starting wavelength is 1546 nm, and the stop wavelength is 1550 nm with a sampling point of 501, hence the sampling wavelength is 0.008 nm.

**Figure 3:**
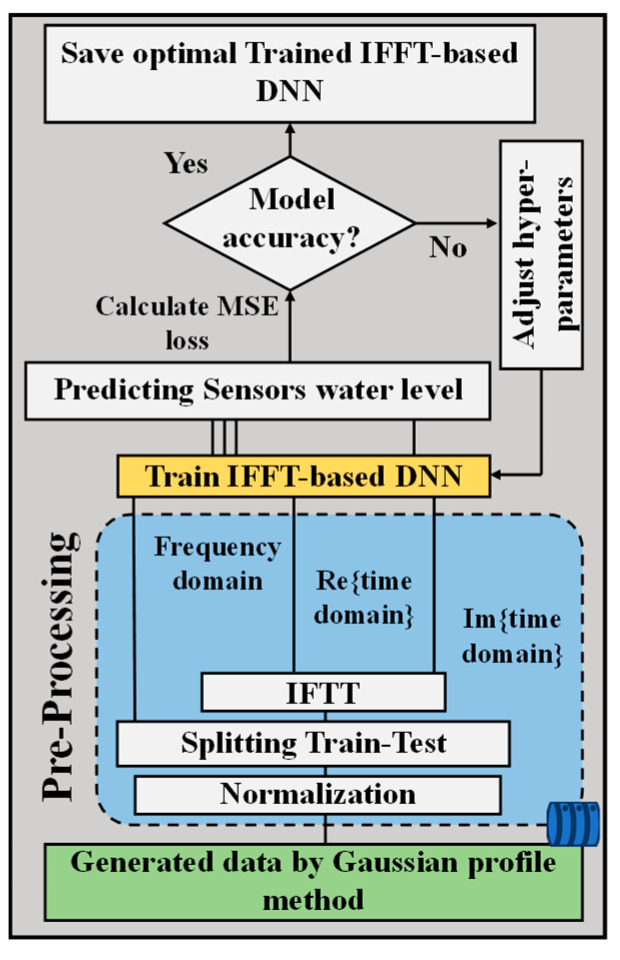
The generate, pre-processing, and water level prediction workflow.

To predict the water level, it is crucial to predict the Bragg wavelength (*λ*B) and then calculate the water level based on the Bragg wavelength. Since conducting numerous experiments to collect data for network training is time-consuming and impractical, the Gaussian profile method is utilized to generate the required data. In this method, the total reflected spectra that are recorded in the OSA can be calculated as follows [16]:

(1) $$R_{\mathrm{total}}(\lambda )=\sum_{i=1}^{n}\sum_{j=1}^{k}\left(I_{\mathrm{peak},i}\times e^{\left(-4\ln2\times {\left(\frac{\lambda -\lambda B_{i,j}}{\Delta \lambda_{\mathrm{FWHM}}}\right)}^{2}\right)}\right)+N(\lambda )$$

where *I*_peak,*i*_ is the peak reflectivity in the *i*th row and Δ*λ*_FWHM_ is the full width at half the maximum intensity of the FBG sensor. The *λB_i,j_* is the center wavelength of *FBG_i,j_* that depends on its water level. The *n* is the number of rows, *k* is the number of sensors in each row, and we consider *N*(*λ*) as a random white Gaussian noise in the system. This method then serves as the initial step of the IFFT-DNN model to produce the required dataset, as depicted in Figure 3. Subsequently, as illustrated in the flow of each state in Figure 3, the generated dataset is normalized, and then an IFFT is applied to each data point. 

The frequency domain input data along with the real and imaginary parts of its time domain, are used as inputs and the corresponding Bragg wavelengths are considered as outputs in the pre-processing stage. This model aims to enhance the accuracy of water level predictions by using a combination of inputs from both the frequency and time domains. To achieve this, X_1_ = *R*_total_(*λ*) is considered as the input in the frequency domain, where *R*_total_(*λ*) represents the total reflected spectrum from the sensors as a function of the wavelength *λ* which is calculated by Equation (1). This input captures the overall response of all the sensors in the system. Additionally, two more inputs derived from the time domain are introduced. The first one is X_2_ = Re{*IFFT*(*R*_total_(*λ*))}, which is the real part of the IFFT of the total reflected spectrum. The second input from the time domain is Im{*IFFT*(*R*_total_(*λ*))}, which is the imaginary part of the IFFT of the total reflected spectrum. The IFFT operation transforms the data from the frequency domain back into the time domain, providing a different perspective on the sensor data. By incorporating both the real and imaginary parts of the IFFT, the model gains a more comprehensive understanding of the time-domain characteristics of the sensor signals. These inputs, X_1_, X_2_, and X_3_, collectively provide a rich set of features from both the frequency and time domains. This dual-domain approach allows our neural network to learn more robust and detailed patterns in the sensor data, improving its ability to accurately predict water levels. The outputs of our model are the Bragg wavelengths, *λ*B_1,_ and *λ*B_2_, corresponding to each sensor, and then the water level is calculated based on them. The Bragg wavelengths shift in response to changes in the water level, so accurately predicting these wavelengths enables us to determine the water levels detected by the sensors.

**Figure 4:**
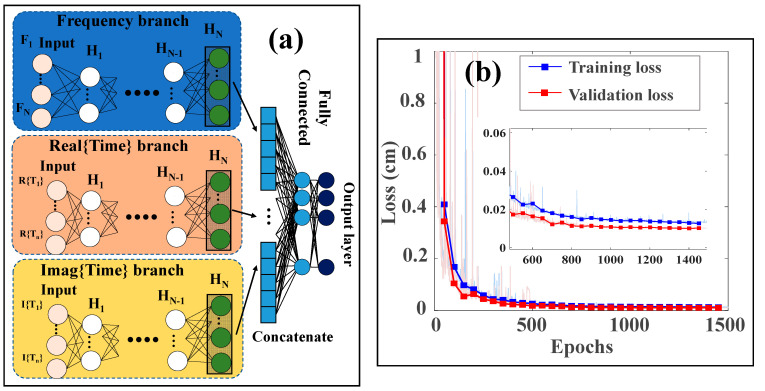
Part (**a**) depicts the IFFT-DNN network and part (**b**) the MSE loss for training and validation set.

For training and validation purposes, we create datasets consisting of input–output pairs: {(X_1_, X_2_, X_3_), (*λ*B_1_, *λ*B_2_)}. Each input set (X_1_, X_2_, X_3_) represents the sensor data in both the frequency and time domains, while the output set (*λ*B_1_, *λ*B_2_) corresponds to the Bragg wavelengths associated with specific water levels. By training the network on these comprehensive datasets, higher accuracy and robustness in water level measurements will be achieved, even in challenging conditions where crosstalk and overlapping may be present. This innovative approach leverages the strengths of both frequency and time domain analyses to enhance the performance of our water level sensing system. Consequently, the generated dataset by the Gaussian profile method is split into 70% for training and 20% for validation during the pre-processing. Afterwards, the IFFT is applied to each Gaussian profile sample in the frequency domain. The frequency domain data, along with the real and imaginary parts of the time domain data for each sample, serve as inputs to the IFFT-DNN for predicting the Bragg wavelength and, subsequently, the water level, as illustrated in Figure 4a.

These three inputs enter the hybrid neural network and traverse multiple hidden layers before reaching the output layer. The hidden layers process various aspects of the input data, including the frequency domain and the real and imaginary parts of the time domain. It is also worth mentioning that dropout and L2 regularization [35,36] are also applied in each branch to prevent overfitting. After processing, the outputs from each branch are concatenated into a unified vector and pass through a fully connected layer to predict the water level. The Mean Squared Error (MSE) is used as the loss function and as the criterion for model accuracy. After training the network, if the desired accuracy is not achieved, the hyperparameters are tuned to improve performance. Once the model reaches its highest accuracy, it is saved and used to predict water levels for the experimental data. As shown in Figure 4b, the network achieves a MSE of less than 0.02 cm for training and validation. Details of the IFFT-DNN network and optimizer settings are provided in Table 1.

## 3. Results and Discussion

**Figure 5:**
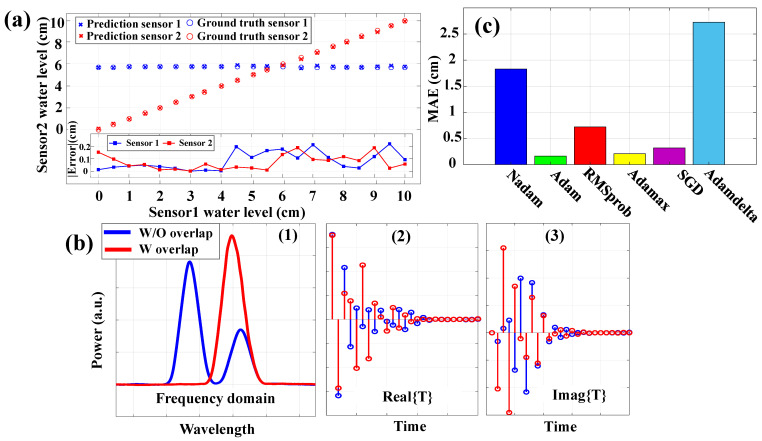
(**a**) The water level prediction and absolute error for each case. (**b**) The inputs of each branch are shown for both overlapping and without overlapping conditions, including the frequency, real part, and imaginary part in time, as illustrated in (1) to (3), respectively. (**c**) The optimizer analyses.

In this section, the performance of the proposed water level sensing system, which incorporates the IFFT-DNN model, is evaluated by using the experimental setup that was previously described in Section 2. The evaluation focused on the system’s ability to accurately predict water levels while there is overlapping and crosstalk between the sensors. The illustrated results in Figure 5a, show that the IFFT-DNN model accurately tracks the true water levels for both sensors while the accuracy is maintained in various experiment cases. The prediction for different water levels of the first and second sensors is shown with blue and red crosses, respectively. The ground truth of each case experiment is shown with blue and red circles, respectively. Furthermore, the inset error graph in Figure 5a confirms that the predictions’ absolute error remains lower than 0.22 cm across the different cases, indicating high precision in the water level predictions model.

In Figure 5b, the three inputs for each branch of the IFFT-DNN model under both overlapping and non-overlapping conditions are depicted. As it can be observed the differences between these conditions in the time domain help the network’s ability to achieve a higher accuracy. A comprehensive analysis to select the optimal optimizer is conducted, with the results illustrated in Figure 5c. The MAE of the experimental results is plotted for various optimizers used in training the IFFT-DNN model. The Adam optimizer demonstrated the best performance, yielding the lowest MAE, followed by RMSprop, Adamax, Nadam, SGD, and Adadelta. Therefore, the choice of optimizer has a substantial impact on both the training efficiency and final prediction accuracy, with Adam emerging as the most effective for this task. It is worth mentioning that both the total error and each case error become much lower than the previous study [37]. In the previous study, a DNN network utilizing only the frequency domain was employed to analyze the sensing system. In contrast, the current manuscript introduces a dual-domain approach, uniquely combining both a frequency and time domain analysis. This dual-domain method has not been extensively investigated in other sensing system research, making this approach novel and innovative.

Furthermore, significant improvements in error reduction have been achieved; while the previous work showed the errors reaching up to 0.5 cm in severe conditions, the current study ensures that each case error does not exceed 0.2 cm. This substantial improvement in accuracy underscores the effectiveness of the dual-domain approach and its potential to set a new standard in fiber optic liquid level sensing systems. By combining the advantages of both frequency and time domain analyses, this study not only reduces the total error but also ensures consistent accuracy across various scenarios, thereby enhancing the robustness and reliability of the sensing system. This dual-domain approach opens up new avenues for other research and represents a significant advancement over previous methodologies.

**Figure 6:**
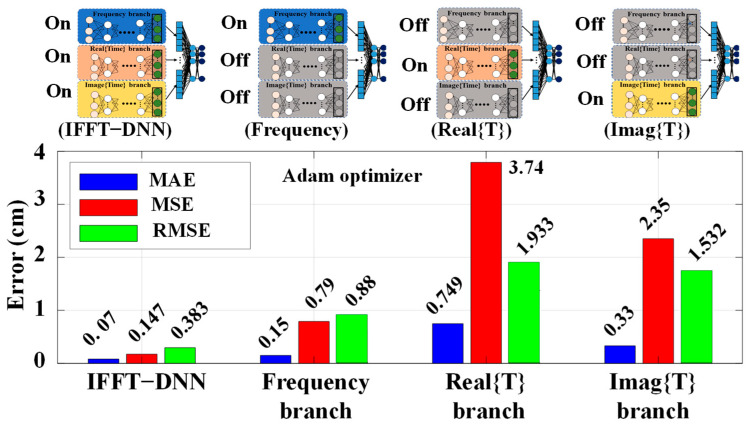
The network performance for each branch versus full IFFT-DNN. The MAE, MSE and RAMS are shown with blue, red and green bars respectively.

The comparative analysis shown in Figure 6 highlights the performance of the full IFFT-DNN model against its branches. The IFFT-DNN model achieves the lowest errors across MAE, MSE, and RMSE metrics, with a MAE of 0.07 cm, which demonstrates its superior performance in predicting water levels. Because the time domain by itself does not directly correlate with the Bragg wavelength, and therefore the water level, it leads to a lower accuracy in the time domain branches compared to the integrated model. Although the individual branches exhibit higher errors, the combination of them will help the network to extract more features and consequently a higher accuracy. The comparison of the full IFFT-DNN model with its individual branches demonstrates the importance of integrating frequency domain features with real and imaginary components of the time domain signal. This integration enhances the model’s feature extraction capabilities, leading to more accurate predictions. As a result, the incorporation of time domain features provides additional information about variations in sensor signals, enhancing the neural network’s ability to accurately predict water levels. As illustrated in Figure 6 and discussed in Figure 5b, the measurements in both domains for overlapping and non-overlapping conditions bring more comprehensive information in different situations to feed the neural network.

Regarding the pre-processing, it should be noted that even with up to a hundred sensors, the pre-processing time does not significantly increase regarding the nature of water level changes which is generally slower than the required pre-processing time. The IFFT-DNN model predicts in only 0.025 milliseconds, which is more than adequate for real-time monitoring. Furthermore, as depicted in Figure 1, several sensors in the same bandwidth operation are pre-processed together, significantly reducing the pre-processing time. The sensors in the experiments were allowed to change independently, demonstrating the system’s ability to handle variations. Controlled linear changes were used to provide clear and understandable scenarios, ensuring that all possible situations were included in the experiment. The simulations also accounted for the more complex scenarios to ensure thorough testing.

**Figure 7:**
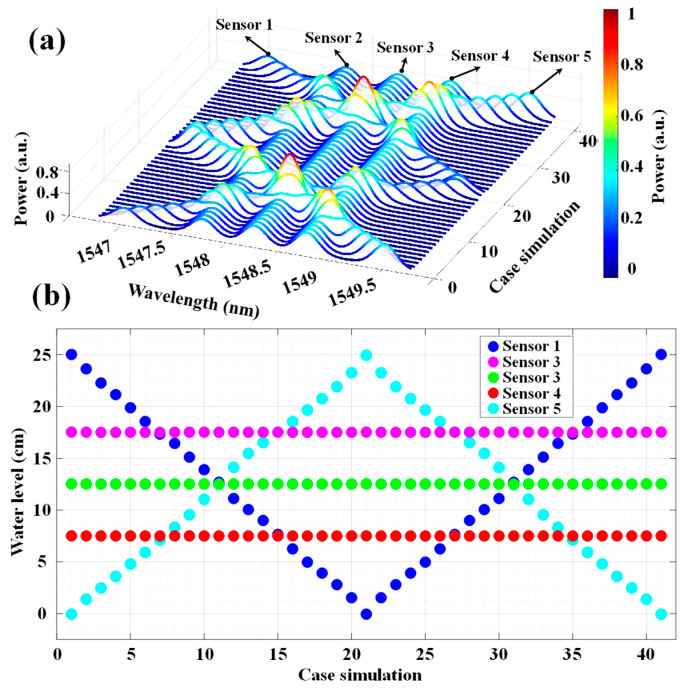
(**a**) The reflected spectrum of five sensors for different case simulations. (**b**) The prediction result of IFFT-DNN for each case simulation.

To further investigate the performance of the proposed method, a simulation was conducted using five water level sensors. The goal was to evaluate the model’s ability to accurately predict water levels under varying conditions. In Figure 7a, the spectrum of the five sensors across 41 case simulations is illustrated, where the water level changes from 0 cm to 25 cm. In this figure, each peak in the power spectrum corresponds to a different sensor, labeled as Sensor 1 through to Sensor 5. The color gradient, ranging from blue to red, indicates the normalized power intensity, with blue representing lower power and red representing higher power. In Figure 7b the IFFT-DNN prediction result is presented with a total MAE of 0.031 cm for all five sensors. The simulation results presented in Figure 7 validate the model’s robustness in handling overlapping signals from multiple sensors. 

Despite the complexities introduced by signal overlap, accurate distinctions and predictions of the water levels for each sensor were achieved. Additionally, the consistency and variation in the measured water levels, as depicted in the results, emphasize the accuracy and reliability of the model in real-world scenarios. This confirms that the dual-domain approach, which incorporates both frequency and time domain features, is effective in enhancing the performance of water level measurement systems. 

Conducting a comparative analysis of various algorithms against the proposed IFFT-DNN method, as shown in Table 2, demonstrates the superior performance of our approach. Specifically, the Mean Absolute Error (MAE) and Mean Squared Error (MSE) metrics were evaluated across four different algorithms: Support Vector Machine (SVM), Random Forest, Deep Neural Network (DNN), and the proposed IFFT-DNN.

## 4. Conclusions

The integration of FSO and the multichannel sensing system with the IFFT-DNN model has demonstrated a highly accurate and efficient solution for remote water level sensing at multiple points. The experiment was conducted with a distance of 2 m and in controlled laboratory conditions for the FSO link and two water level sensors. The experiment results highlight that the system has a low MAE of 0.07 cm for predicting water levels even under the challenging conditions of signal overlapping and crosstalk. Additionally, the simulation result for a greater number of sensors shows a MAE of 0.031 cm which approves the robustness of the proposed method. The use of FSO technology significantly enhances the sensing system by enabling high-speed data transmission over long distances without the need for physical cables, which not only simplifies deployment but also reduces installation costs, and makes it an economically viable option for remote applications. Additionally, the robustness of the IFFT-DNN model in extracting features from both the frequency and time domains ensures accurate water level measurements, even in complex scenarios with overlapping signals.

## Figures and Tables

**Table 1:** The details of IFFT-DNN.

Parameter	Value	Parameter	Value
Hidden layers (per branch)	11	Activation	ELU
Neurons per Layer	95	FC1 Neurons	250
L2 Regularization	10^−5^	FC2 Neurons	100
Dropout Rate	0.009	Batch Size	368
Input Size	3 × 501	Loss Function	MSE
Output Size	2	Optimizer	Adam
Decay rate	0.96	Decay step	800
Epochs	1500	Decay rate	0.96
Learning Rate Decay	Exponential	Initial Learning Rate	0.003

**Table 2:** Comparing IFFT-DNN with other algorithms.

Algorithm	MAE	MSE
Support vector machine	0.6 cm	2.03 cm
Random forest	0.48 cm	1.76 cm
DNN	0.15 cm	0.79 cm
IFFT-DNN	0.07 cm	0.147 cm

## Data Availability

The data presented in this study are available on request from the corresponding author.
